# Intelligent Control Wheelchair Using a New Visual Joystick

**DOI:** 10.1155/2018/6083565

**Published:** 2018-02-07

**Authors:** Yassine Rabhi, Makrem Mrabet, Farhat Fnaiech

**Affiliations:** Laboratoire SIME, Ecole Nationale Supérieure d'Ingénieurs de Tunis (ENSIT), Université de Tunis, 5 Av. Taha Hussein, 1008 Tunis, Tunisia

## Abstract

A new control system of a hand gesture-controlled wheelchair (EWC) is proposed. This smart control device is suitable for a large number of patients who cannot manipulate a standard joystick wheelchair. The movement control system uses a camera fixed on the wheelchair. The patient's hand movements are recognized using a visual recognition algorithm and artificial intelligence software; the derived corresponding signals are thus used to control the EWC in real time. One of the main features of this control technique is that it allows the patient to drive the wheelchair with a variable speed similar to that of a standard joystick. The designed device “hand gesture-controlled wheelchair” is performed at low cost and has been tested on real patients and exhibits good results. Before testing the proposed control device, we have created a three-dimensional environment simulator to test its performances with extreme security. These tests were performed on real patients with diverse hand pathologies in Mohamed Kassab National Institute of Orthopedics, Physical and Functional Rehabilitation Hospital of Tunis, and the validity of this intelligent control system had been proved.

## 1. Introduction

With the large increase in the number of older people and people with physical difficulties, there are significant applications for the navigation assistance of intelligent wheelchairs. Due to accidents, elderliness, or diseases as cerebral palsy and spinal cord injuries, the proportion of disabled people is rising up and now representing 1 billion persons, which represent 15% of the global population [1].

**Figure 1:**
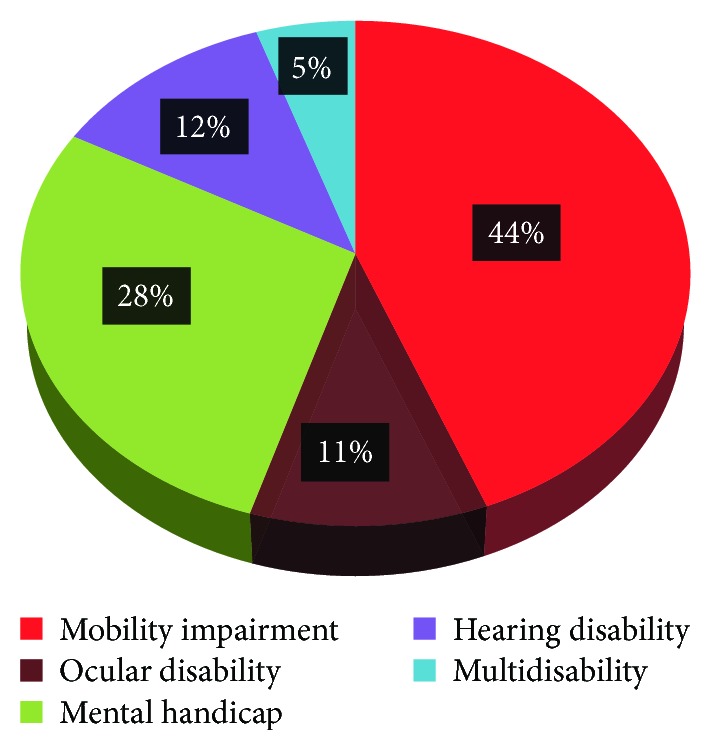
The distribution of handicap prevalence in Tunisia in 2013.

Moreover, according to the Tunisian statistic study from the Ministry of Social Affairs of Tunisia 2013 published in an online source, more than 208,465 Tunisians suffer from variant disabilities, where they represent 2% of the entire population.  Figure 1 shows the distribution of this handicap prevalence.

We recognize many types of physical disabilities. They occur under different aspects like ataxia, spasticity, or motor dysfunction, which cause a lack of muscle coordination, involuntary movements, a delay in reaching motor skills, shaking, tremor, and the inability to control the movements especially precise ones like writing. These will obviously cause a lack of independent mobility, self-esteem, and safety that requires the use of adaptive equipment such as a manual wheelchair and electric wheelchair or the help of a caregiver to do their daily life activities. Obviously, the electric wheelchairs were the best-proposed solution. However, many maneuvering difficulties occur with people suffering from upper extremity impairments. They do not have the power to properly manipulate their electric wheelchairs, neither do they have the reflex to rapidly decrease velocity in serious situations. Consequently, a conventional powered wheelchair manipulated via a joystick does not fulfill their requirements. A clinical survey has been presented in [2]. First, it has shown that 10% of the patients cannot use the electric wheelchair in daily life activities. Second, 40% of regular powered wheelchair users have difficulties with steering tasks such as passing through open doors, and nearly 9% of them find it impossible without assistance. Then, 18% to 26% of nonambulatory patients who cannot use a manual wheelchair cannot use a powered wheelchair.

To accommodate those mobility-impaired persons, numerous cutting-edge techniques and functionalities have been developed over the last years. The trend of increasing intelligence has been encouraged by low-cost processors and sensors. For these reasons, many researchers have been working to find new, sophisticated control algorithms for easy handling of the wheelchair during the last 20 years.

Indeed, many works based on wheelchairs have been proposed to improve its usability. The human interface for easy operation of the intelligent wheelchair is the most popular research issue. In [3], the authors propose a novel method to classify human facial movement based on multichannel forehead biosignals. A novel hands-free control system for intelligent wheelchairs based on visual recognition of head gestures is used in [4–7]. Hence, in [8–10], the authors developed a voice-controlled wheelchair. In [11, 12], the authors use the sip-and-puff (SNP) system. In this system, the user either draws in air or blows air into a wand or tube. This system requires calibration for each user; once calibrated, it will only recognize the specific user's sips and puffs. The SNP system recognizes four different commands, hard sip, soft sip, hard puff, and soft puff. In [13, 14], the patient controls the wheelchair by tracking the eyes for blinks and estimating gaze direction through a camera placed in front of the wheelchair users. A different and newer ideal is the Tongue Drive System (TDS) developed by a team at Georgia Tech [15]. This system uses two magnetic sensors placed on the side of the users' head and magnetic tongue barbell. In [16], the brain-computer interface (BCI) is a direct communication between the brain and the computer, where a set of electrodes attached to the scalp collects brain signals and transfers them to a computer.

All the interfaces mentioned above have predetermined commands to move the wheelchair. It allows only four different commands (forward, backward, left, and right) at a predefined speed.

In addition, these control techniques are too tedious for the patient when he/she has to drive for a long time. Indeed, the patient should blink his/her eye and make facial gestures across his/her path.

The wheelchair control based on hand movement tracking has achieved a great success in recent years [17–28]. In this work, we briefly examine some previous work on this subject to put our work in the context. Hand movement is actually done in a 3D space. Then, hand tracking may be performed in a 3D space or in the 2D image plane as required. That is why hand tracking approaches can be classified in 2D and 3D methods. In the 2D space, the hand is characterized by its geometric form such as edges [17, 25] and the finger shapes [18]. We found nongeometric characteristics like color and texture [19]. Many works have been presented in the literature. Isard and Blake described in [26] an approach for hand tracking by skin-colored blob tracking. The authors in [22] recently proposed to incorporate the optical flow and color cues for fast hand tracking. Hand tracking in a 2D space is very effective for real-time applications. Therefore, many applications were based on 2D methods [27]. However, the 2D tracking cannot translate all gestures made by the hand. Consequently, 3D hand tracking allows the location in the 3D space and extracts the 3D position and orientation. During our work, we found many works that used 3D hand tracking [20, 21, 23, 24, 28]. Although 3D methods provide more accurate results than 2D tracking, they usually suffer from high computational cost. Thus, the 3D tracking is rarely used in real-time applications.

This work is addressed to people with severe upper extremity impairments; we propose to them an intelligent wheelchair's joystick control system allowing them to move toward the desired point with a suitable acceleration. The system uses visual hand gestures to control and to assist variable speed navigation (like navigation with an analog joystick). This proposed control system presents an efficient hands-free option that does not require sensors or contraptions attached to the user's body or special camera on the wheelchair.

**Figure 2:**
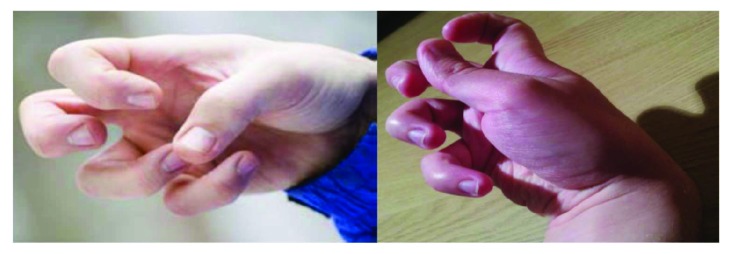
The hand posture of a person suffering from dystonia.

In the case of our target users, this modality appears to be very suitable: this visual interface can be handled even with a cramped posture of the hands (Figure 2). In addition, the use of the proposed interface requires less muscular effort than that of a joystick.

For this control mode, our proposal is to develop a robust control system with the visual recognition of the hand movement for patients with problems of physical accessibility in the 2D image plane. Firstly, we are going to focus on recognition movement through hand features. Second, extract approaches in order to realize a real-time control application that detects the hand of the user through a video and finally extract the features and analyze them in order to detect his/her current position state. To solve user limitations, we integrated into the visual joystick the behavior calibration system that shares the control with the user and assures the suitable command with a real-time support. This calibration system is based on an artificial intelligence method. Therefore, a neural network algorithm is then applied; it is currently used for both research and production by different teams in many applications in medicine, engineering, and so on. The designed system is an intelligent controller that corrects constantly undesirable movement of the hand and assures a smooth and safe navigation, respectively, with variable speed navigation. All the tests using the artificial intelligence algorithm are performed in real time on a large database of collected frames in a wide variety of conditions. A three-dimensional simulation environment is then designed to test the performances of the new proposed system.

In the next section, the proposed methodology to create the smart wheelchair is described. Experimental results are presented in Section 2. The last section concludes this work.

## 2. Methods

In the introduction, some control techniques have been reviewed. In practice, these techniques were implemented on real wheelchairs. For instance, for severely disabled people, the most appropriate technique would be the smart one. However, this type of intelligent wheelchair presents various technical and psychological problems.

From a technical point of view, to design a fully reliable and robust automatic intelligent wheelchair, we must use sophisticated sensors to detect perfectly the environment and to implement a real-time effective algorithm providing suitable real and safe decision. Unfortunately, until now, the sensors are still expensive and the used algorithms for automatic navigation are too heavy and time consuming.

From the psychological point of view, many potential users, in the beginning, are mistrustful to give the total motion control of their wheelchair. Always, the user prefers to be the main decider and the main driver of his/her own movement.

### 2.1. Architecture of the New Control System

We aim to develop a hand gesture control law of the wheelchair based on recurrent neural networks. This law uses the information on rehabilitation and learning sessions to provide an intelligent system navigation that takes into account the nature of the disability.

**Figure 3:**
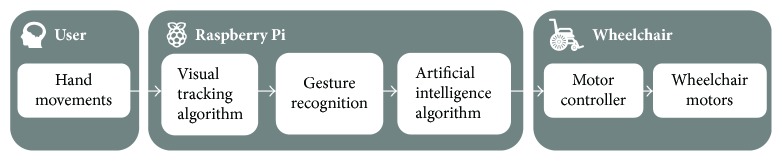
Proposed system architecture.

The global architecture of the intelligent assistance system and the connection between its elements are presented in Figure 3. In the next step, we explain in detail each of these elements.

The first step is the acquisition of the hand movement by a visual sensor (USB-camera). The patient is asked to put his/her hand in front of the camera to catch an image from the video. The second step is to receive a signal in real time and displays live video streaming of the hand motion of the patient sitting on the wheelchair. Then, the proposed smart interface allows the user to easily control their wheelchairs directly by changing the position of the hand. Since some patients cannot move their hand in the right directions, we will integrate the recurrent neural network to make the necessary corrections. In the final step, we inject the suitable outputs into the electrical wheelchair.

### 2.2. Approach

Our application is based primarily on computer vision, image processing, and pixel manipulation, for which there exists an open-source library named OpenCV (Open Source Computer Vision Library), consisting of more than 2500 optimized algorithms. OpenCV uses an algorithm of facial recognition; object analysis and identification; human gesture classifications in videos, achieved with filters; edge mapping; image transformations; detailed feature analysis; and more other operations.

These functions provided by this library are also essential in the development process of the hand tracking application.

The focus is set on using the frames from a live camera feed. The image thresholding is provided using an HSV color space. Finding the segmented areas is done based on their detected edges and the centroid's computation. Thus, changing parameters is done during runtime.

#### 2.2.1. Hand Detector and Background Subtraction

The first step to realizing our goal of the visual control is the tracking of the user's hand. The most used solution is the segmentation of the hand from the background. One of the extremely used characteristics to detect the hand is the skin color as it has been proven to be an efficient feature [29, 30].

**Figure 4:**
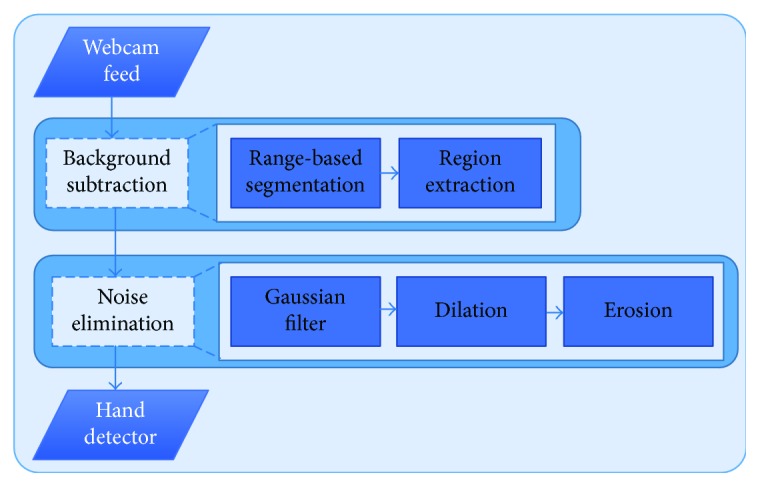
Hand detector procedure.

As illustrated by the flowchart in Figure 4, our task in this step is to segment the hand in the image from the background. Skin color detection aims at determining whether a color pixel has the color of human skin or not. This type of classification should overcome difficulties such as different skin tones (white, pink, yellow, brown, and black), scene illuminations, and the fact that background pixels can have the same color as those of skin [15]. The problem of the RGB color space is that it does not provide the correct information about skin color due to the problem of luminance effects. HSV provides color information as hue (or color depth), saturation (or color purity), and intensity of the value (or color brightness). Hue (H) refers to the color of red, blue, and yellow and has the range of 0 to 360. Saturation (S) means the purity of the color and takes the value from 0 to 100%. Value (V) refers to the brightness of the color and provides the achromatic idea of the color. From this color space, H and S will provide the necessary information about the skin color.

Recall that the *RGB* to *HSV* transformation can be expressed as

(1) $$\begin{array}{c} HSV\left\{\begin{array}{c} H=\left\{\begin{array}{ll} h,B\leq G,\\ 2\pi -h,B>G, & \;h=\cos^{-1}\frac{1/2\left[\left(R-G\right)+\left(R-B\right)\right]}{\sqrt{{\left(R-G\right)}^{2}+\left(R-G\right)\left(G-B\right)}}, \end{array}\right.\\ S=\frac{\max\left(R,G,B\right)-\min\left(R,G,B\right)}{\max\left(R,G,B\right)},\\ V=\max\left(R,G,B\right). \end{array}\right. \end{array}$$

**Figure 5:**
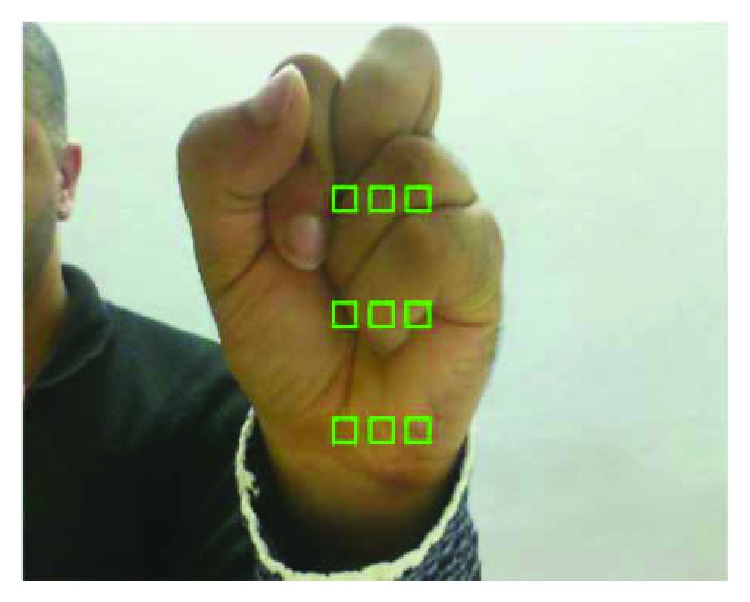
Hand detector procedure.

Initially, we present a set of fixed windows in the webcam feed. The user is asked to put his/her hand close to the screen to cover the 9 green squares to generate the hand color data to the program (as shown in Figure 5). It is important to mention that the square number is an empirical value and it can vary.

After the 9 hand color data are obtained, 9 upper and lower boundaries for the hand area are computed. Then, we calculate the averages of these boundaries in the HSV color space, which can be represented as a 6-dimensional vector (Color Profiler). The bounding vector is automatically generated by the program. The Color Profiler is used to find binary segmentation of the hand from the background. Therefore, the segmentation performance actually depends on the choice of the bounding vector.

The following steps show how to find the hand skin color.

**Figure 6:**
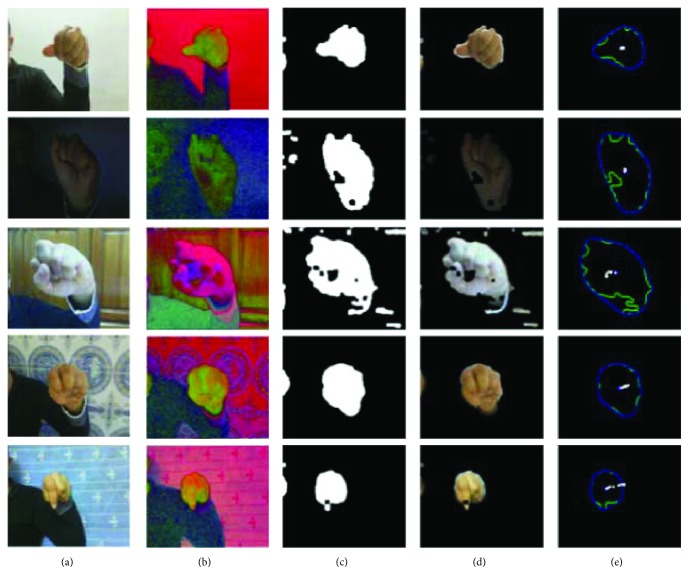
Real-time hand detection in complex backgrounds under various lighting conditions.

1. Convert the image in Figure 6(a) to the HSV color space in Figure 6(b).
2. Throw away the V channel and consider the H and S channels and hence discount for lighting variations.
3. Threshold pixels with low saturation due to their instability.
4. Bin the selected skin region into a 2D histogram. This histogram now acts as a model for the skin.
5. Compute the “back projection” “(i.e., use the histogram to compute the “probability” that each pixel in our frame has the color of the skin tone).
6. Skin regions will have high values.

In this case, the resultant image was segmented to get a binary image of the hand. Binary images are two-level images where each pixel is stored as a single bit (0 or 1). Smoothening was needed, as the output image had some jagged edges as clearly seen in Figure 6(c). There can be some noise in the filtered images due to false detected skin pixels or some skin color objects (like wood) in the background; it can generate few unwanted spots in the output image as shown in Figure 6(e).

Firstly, we created an elliptical structuring kernel which will be used to perform three iterations of erosions and dilations, respectively [31]. These operations will help us remove the small false skin regions in the streaming. Using dilation and erosion results in a morphological closing causes the light regions to join together and therefore improves the detection.

Thereafter, a Gaussian filter (2) (size: 3 × 3) is used for smoothing and noise elimination in order to improve the image quality for better interpretation as follows:

(2) $$\begin{array}{c} G\left(x,y\right)=\frac{1}{2{\pi \sigma }^{2}}e^{-\left(\left(x^{2}+y^{2}\right)/2\sigma^{2}\right)}. \end{array}$$

Several tests were conducted in different lighting conditions and different backgrounds, as can be seen in Figure 6.

Once the image is segmented, the hand contour is found. For this, we need to eliminate all the blobs other than the hand, segmented with respect to the background. The BLOB (Binary Linked Object) method is then applied. The remaining contour corresponds only to the hand of the largest contour in the frame as shown in Figure 6(e).

#### 2.2.2. Hand Tracker

*(1) Standard Hand Tracking*. Once the image is segmented, the hand contour is obtained. The next step involves finding the largest closed contour in this image. Then, the area center is calculated.

**Figure 7:**
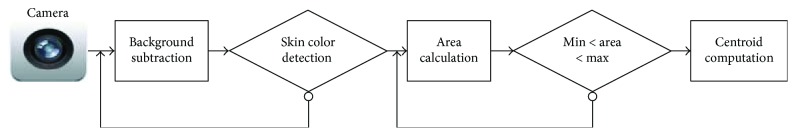
Extraction of hand coordinates.

After collecting information about all segmented areas, the size and the central coordinates of these areas within each contour are determined. Note that these contour-bound areas are considered objects if their surface value does not exceed the predefined minimum value and remains below the maximum.  Figure 7 shows the steps to follow.

Let *I*(*x*,*y*) be the pixel value at the hand position (*x*,*y*) in the 2D image. The zero-order moment is given by (3). The order spatial moments and the center of mass are given by (4) and (5). The zero-order moment represents the area occupied by the shape of the frame.

(3) $$\begin{array}{c} M_{00}=\sum_{x}\sum_{y}I\left(x,y\right). \end{array}$$

The one-order moments (*M*_10_, *M*_01_) are calculated by

(4) $$\begin{array}{c} M_{10}=\sum_{x}\sum_{y}x\times I\left(x,y\right),\\ M_{01}=\sum_{x}\sum_{y}y\times I\left(x,y\right). \end{array}$$

The center of mass of the hand is computed using the zero-order and one-order moments, which (*x_c_*, *y_c_*) are presented by

(5) $$\begin{array}{c} x_{c}=\frac{M_{10}}{M_{00}},\\ y_{c}=\frac{M_{01}}{M_{00}}. \end{array}$$

**Figure 8:**
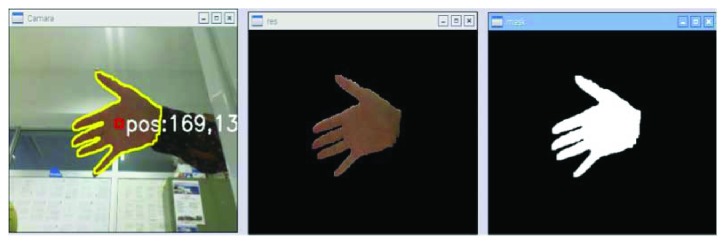
Screenshot of the hand with the center of mass marked with a red rectangle.

Figure 8 shows the results of the computation of the center of mass of the hand (red rectangle).

*(2) Hand Tracking with the Kalman Filter*. Finding the region of interest (ROI) with repeated detection of the hand gives a very noisy tracking. The Kalman filter that estimates and smooths each frame of the hand position sequence is applied [32, 33]. The input parameters of the Kalman filter are the positions of the hand in the image at time *k*. This filter can accurately smooth and predict the position and the velocity to reach the target. Thus, the Kalman filter is used to estimate and to smooth the position of the hand in motion. The state equation of the system is given by (6) and the observation equation by (7).

(6) $$\begin{array}{c} X_{k+1}=A_{k}X_{k}+W_{k}, \end{array}$$

(7) $$\begin{array}{c} Y_{k}=H_{k}X_{k}+V_{k}, \end{array}$$

where **X***_k_* is the state vector of the system, *X*_*k*+1_ is the next state, **A***_k_* is the state transition matrix, *Y_k_* is the measurement state of the system, **H***_k_* is the observation matrix, *W_k_* is the dynamic noise corresponding to the state vector, and *V_k_* is the measurement noise corresponding to the observation vector.

(8) $$\begin{array}{c} X_{k}={\left[x_{k}\;y_{k}\;{\dot{x}}_{k}\;{\dot{y}}_{k}\right]}^{T},\\ Y_{k}={\left[\begin{array}{cc} x_{k} & \;y_{k} \end{array}\right]}^{T}. \end{array}$$

The estimation vector is

(9) $$\begin{array}{c} X_{k}^{\prime }={\left[\begin{array}{llll} x_{k}^{\prime } & y_{k}^{\prime } & {\dot{x}}_{k}^{\prime } & {\dot{y}}_{k}^{\prime } \end{array}\right]}^{T}, \end{array}$$

where $x_{k}^{\prime },y_{k}^{\prime },{\dot{x}}_{k}^{\prime },\text{and }{\dot{y}}_{k}^{\prime }$ are the position and velocity of the target predicted by the Kalman filter.

From (6) and (7), we can obtain the state transition matrix **A** and the observation matrix **H**, respectively.

(10) $$\begin{array}{c} A=\left[\begin{array}{llll} 1 & 0 & dt & 0\\ 0 & 1 & 0 & dt\\ 0 & 0 & 1 & 0\\ 0 & 0 & 0 & 1 \end{array}\right],\\ H=\left[\begin{array}{ll} 1 & 0\\ 0 & 1\\ 0 & 0\\ 0 & 0 \end{array}\right], \end{array}$$

where dt is the difference between the two moments *k* and *k* + 1 (dt = 1).

*W_k_* and *V_k_* are assumed to be jointly independent white noises; they are also assumed to be independent of the state vectors and of the measurements. Normal and white distributions are presented by

(11) $$\begin{array}{c} p\left(w\right)∼N\left(0,Q\right),\\ p\left(v\right)∼N\left(0,R\right). \end{array}$$

### 2.3. The Proposed Visual Joystick

As previously mentioned, our goal is to replace the conventional joystick of the electric wheelchair by a new visual smart control device. It takes into consideration the altered musculoskeletal hand and the movement disorders.

**Figure 9:**
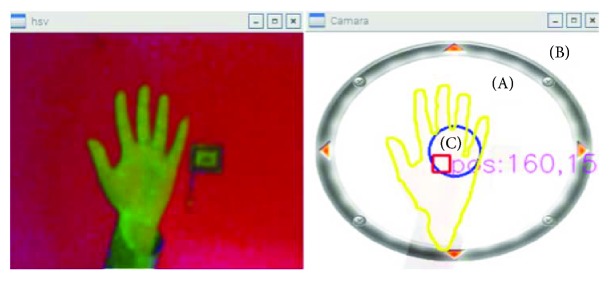
The composition of the visual joystick.

The design of the visual joystick is done by matching the gravity center of mass of the hand (calculated above) to the center of the joystick and then adjusting the joystick axis values in the range (−1, 1). The visual joystick is presented in Figure 9 where the area (A) is reserved to the background to which we can add animation or display some information such as speed movement, acceleration, and time. (B) denotes the area outside the contour of the working area of the visual joystick. Ultimately, the last part (C) is considered the dead area. Hence, the movement in this area of the joystick is considered halted. During navigation, we use the smoothing mode, and when smoothing is enabled, the joystick is reset slowly in the start position if the hand is not in the position.

The coordinates of the center of the hand are related to the origin point by a vector characterized by a distance *r* and an angle *θ*, where *r* is the speed of displacement and *θ* is the rotation angle. These polar coordinate movements are computed as follows:

(12) $$\begin{array}{c} r=\sqrt{{\left(a-x\right)}^{2}+{\left(b-y\right)}^{2}},\\ \theta =\tan^{-1}\left(\frac{y-b}{x-a}\right). \end{array}$$

**Figure 10:**
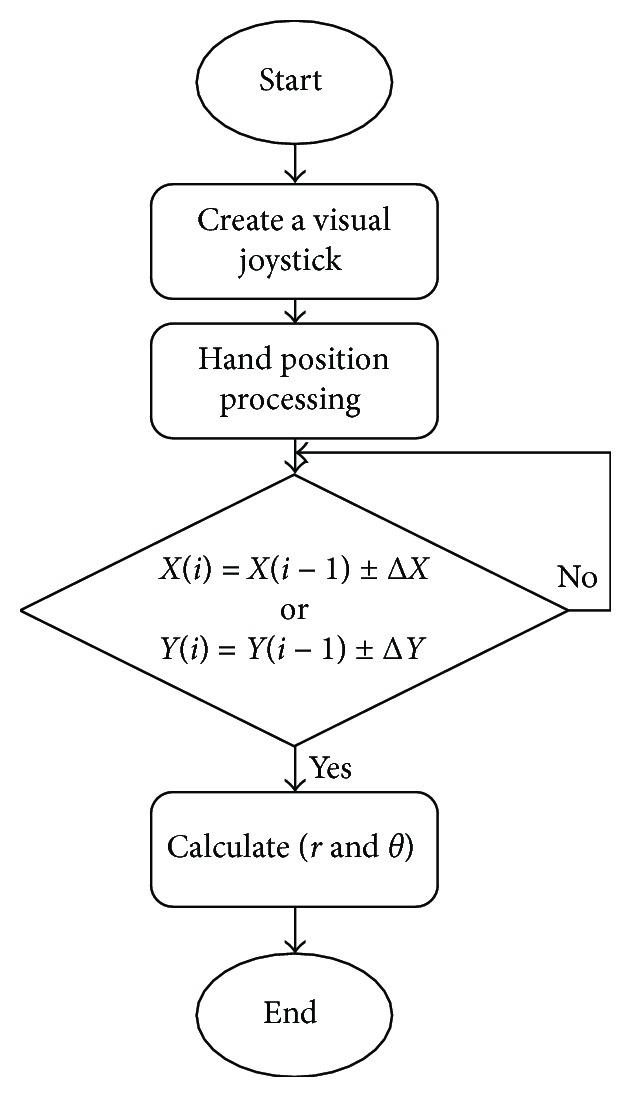
The flow chart of the proposed joystick.

The creation of the new visual joystick is described in Figure 10.

A simple low-pass filter can filter a small hand movement; it may also be programmed with a variety of customized algorithms or other filters. In this step, our objective is to filter small movements without adding a significant delay. We customized the Δ*X* and Δ*Y* present in the flow chart (Figure 10) for each subject. These values involve setting a dead zone and bias axes and establishing optimal gain that could potentially improve control interfaces for people with tremor. It has been validated during the virtual driving tasks when subjects sat in their own wheelchairs. Improving a visual joystick use has application for not only the wheelchair but also computer access, augmentative communication, automobile driving, and environmental control.

### 2.4. The Smart Visual Joystick

In practice, to maneuver a standard joystick, the user must exert predetermined forces to move the wheelchair. In this work, a visual gesture recognition system is built. The control parameters (*r*, *θ*) of speed and angular position of the wheelchair are computed for driving tasks. Hence, the only effort required from the user is to indicate the direction of the wheelchair motion by his/her hand.

The first task for a gesture recognition system, which is equipped with a camera, is a calibration algorithm, mainly for the physically disabled people such as those with quadriplegics and muscular dystrophies, elderly who have muscle weakness, or Parkinson's disease patients who no longer have voluntary movements of the limbs. Calibration is necessary because it is difficult to perfectly align a camera's coordinates to the control system behind it. Thus, a calibration algorithm was developed after identifying the sources of visual joystick errors. Several sources of error affect the coordinates *r* and *θ* generated by the proposed joystick. The pathological body state of a patient, image noise, and scaling factors are the most important sources of error. Any of these errors can involve incorrect data. Hence, it needs to be recovered.

All experiments were performed in our laboratory during the daytime and nighttime. Subjects were seated in front of a computer monitor and moved their hand in front of the camera (the camera is 30 cm away from the patient). They achieved an out-and-back aiming task by moving the hand to match the cursor with the target. The cursor, which is the visual feedback of the position of the hand, was displayed on the computer monitor in the form of a red rectangle.

**Figure 11:**
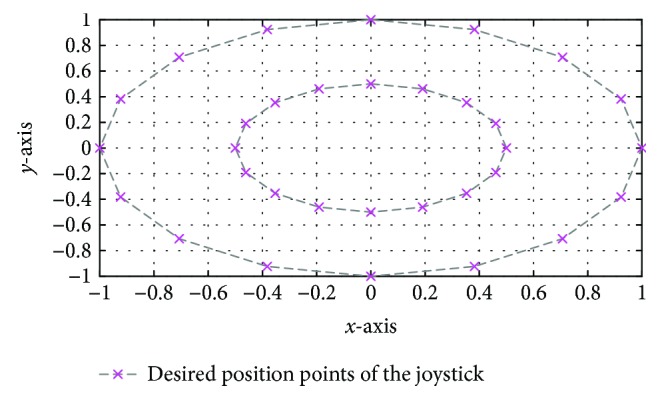
Desired position points of the joystick.

The target was a pink cross (4 mm diameter) displayed on the screen. Each trial started with the target (in pink) and the cursor (in red) in the center of the monitor. Then, this target jumps from the screen center to another position randomly selected from 33 positions. The objective of this test is to scan all the areas that can be reached by the patient while following the reference positions (Figure 11). The target (pink) remained at its new position for 10 s. This test is repeated 10 times to collect all possible positions for each desired position. Each test can take between 5 and 5.5 min.

**Figure 12:**
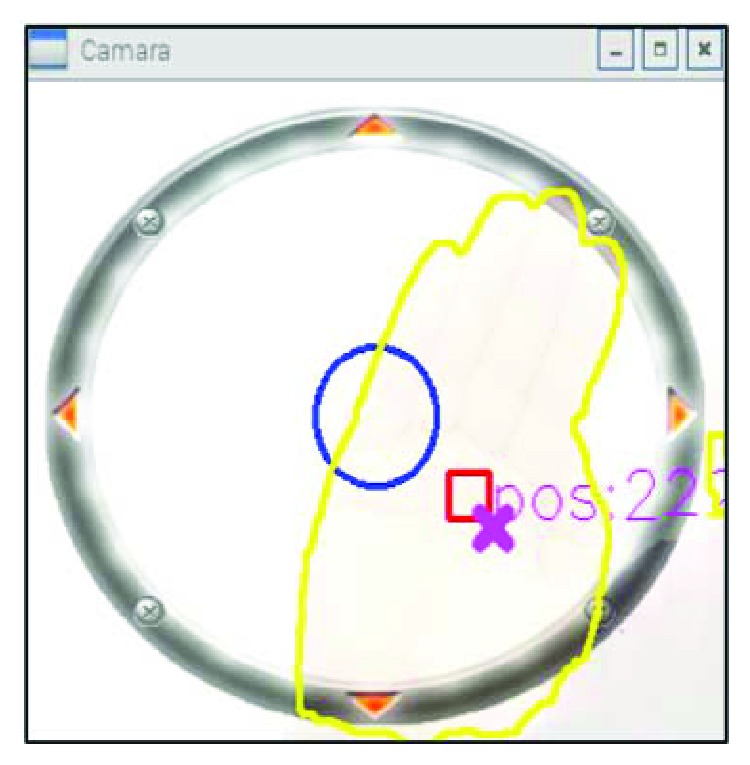
The superposition of the user's visual joystick and the desired position.

During each test session, the subjects have to keep trying to move her/his hand in order to fit the red cursor with the target pink cross despite some muscular disturbances. Such troubles can cause angular rotation between the target position and the actual movement of the hand. Therefore, the cursor, that is, the visual feedback of the hand position, was deviated from its actual position (Figure 12).

As we previously mentioned, the calibration algorithm was developed after identifying the sources of hand movement errors from the database (desired coordinates *r* and *θ* and disability motion).

In fact, we added an artificial intelligence algorithm to the visual joystick. It aims to create a smart controller, which is able to control and correct the existing problems during wheelchair maneuvering.

Recurrent neural network algorithms (RNN) are used to control wheelchairs. Because of their performances, this tool is widely used in this field. In [34], the user's speech is used to train the recurrent neural network for a wheelchair control based on speech recognition. An intelligent neural joystick that eliminates the effects of hand tremor has been implemented [35].

In our case, the collected data (ideal displacement and displacement of handicap) are introduced to a recurrent neural network learning algorithm, in order to estimate the optimal recurrent neural network that modifies the different errors appearing during the driving test.

**Figure 13:**
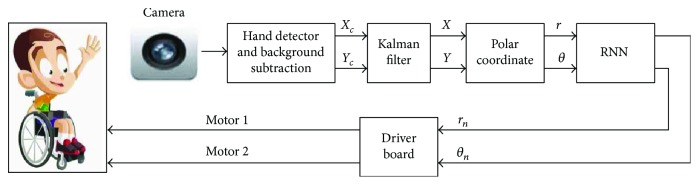
Synoptic of the smart visual joystick.

Finally, we implement this recurrent neural network in the proposed visual joystick algorithm, which allows each disabled person to have his/her specific smart visual joystick.  Figure 13 describes the various steps to make the joystick intelligent.

**Figure 14:**
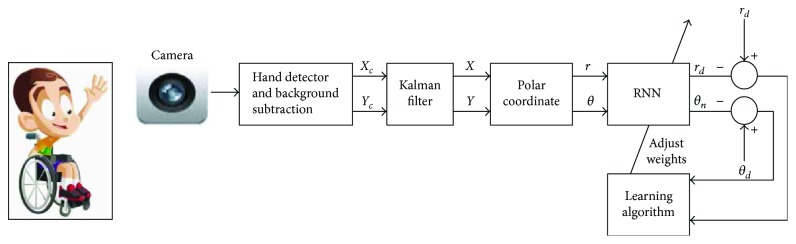
The training step of the visual joystick.

The training set of the recurrent neural network is composed of a couple of data which are the desired positions represented by the vector (**r***_d_*, θ*_d_*) and the corresponding position given by the patient represented by the vector (**r**, θ) (Figure 14).

The model of the RNN training is shown in Figure 14. It has several layers of information beginning with an input layer. In this layer, normalized feature data is forwarded to the model.

The output layer consists of two nodes that give the predicted and the corrected data (*r_n_* and *θ_n_*). We used one hidden layer. Weight adaptation is done with a hyperbolic tangent sigmoid transfer function. All layers have biases. The training is provided by minimizing the mean square error (MSE).

A supervised feed-forward algorithm was proposed. Furthermore, the number of hidden layers and the number of the nodes are chosen according to the cross-validation method to select the proposed optimum RNN structure.

The training-bearing data set is divided into five equally sized subdatasets (five folds). Then, five iterations of training and validation are performed. Each iteration presents four folds for the training and one fold for the validation. In addition, fivefold cross-validation experiments are done to select the optimal number of hidden layers and node number in each layer. Finally, the training is stopped when the error is lower or equal to a fixed MSE.

In order to test our new smart virtual joystick without damaging the patient, we have developed a 3D simulator system that will be well described in the next section.

### 2.5. 3D Virtual Simulator

The development of a motion simulation is capable of simulating the wheelchair navigation in a virtual environment and may be beneficial for

- optimization of the wheelchair designs;
- users' training on electric wheelchair operation;
- users' training on assistive technologies used with electric wheelchairs;
- evaluating, testing, and designing assistive technologies;
- architectural route planning and accessibility studies.

The use of virtual reality in the rehabilitation in medical conditions was suggested as early as 1998 [36]. For 15 years, virtual reality-based systems have been developed to address cognitive, motor, and behavioral disabilities in the assessment areas, rehabilitation and training [37, 38]. Rehabilitation in virtual reality offers the opportunity to bring the complexity of the real world into a controlled environment available in the laboratory [39]. Based on various physical variables that influence behavior while recording physiological and kinematic responses, the virtual reality system grants to design a synthetic environment [40]. Several kinds of simulators are used in the rehabilitation field [41–43].

There are numerous constructors of electric wheelchairs in the world. They aim to overcome disabilities encountered by users [44]. The first wheelchair was developed in the early 17th century with two-wheel drive and two swivel wheels. The motion equations are almost the same for most manufacturers of electric wheelchairs [45].

**Figure 15:**
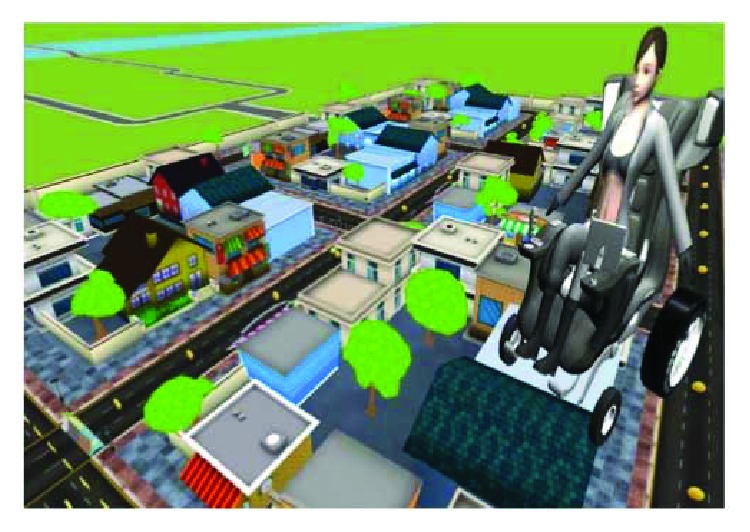
Virtual environment.

To ensure that our virtual simulator (Figure 15) is close to the reality, we used the kinematics and dynamic modeling of our electric wheelchair.

The linear speed of the electric wheelchair at the middle of the two driving wheels is formulated as follows:

(13) $$\begin{array}{c} V_{cw}=\frac{1}{2}\left(V_{L}+V_{R}\right). \end{array}$$

The rotation speed, in the center point between the two driving wheels of the wheelchair, can be calculated using

(14) $$\begin{array}{c} \omega_{cw}=\frac{1}{\lambda_{s}}\left(V_{L}+V_{R}\right), \end{array}$$

where *λ*_s_ is the length of the shaft between the two driving wheels and *V*_L_ and *V*_R_ are the left and right velocities of the driving wheels. The kinematic model of the chair can be determined as follows:

(15) $$\begin{array}{c} \left[\begin{array}{c} \dot{x}\\ \dot{y}\\ \dot{\psi } \end{array}\right]=\left[\begin{array}{ccc} \cos\psi & -\sin\psi & 0\\ \sin\psi & \cos\psi & 0\\ 0 & 0 & 1 \end{array}\right]\left[\begin{array}{c} V_{cw}\\ 0\\ \omega_{cw} \end{array}\right], \end{array}$$

where $\dot{x}$ and $\dot{y}$ represent the speed over (*x,y*) of the wheelchair and $\dot{\psi }$ is the displacement angular velocity.

The dynamic modeling of the wheelchair is presented by

(16) $$\begin{array}{c} F_{L}+F_{R}-m_{cg}\sin\theta =m_{cg}\alpha_{cw},\\ \frac{\lambda_{s}}{2}F_{L}-\frac{\lambda_{s}}{2}F_{R}=J_{cg}{\dot{\omega }}_{cw},\\ \alpha_{cw}={\dot{V}}_{cw}, \end{array}$$

where *θ* is the wheelchair's heel angle with respect to the ground level and *F*_L_ and *F*_R_ are the left and right forces applied by the driving wheels, respectively.

(17) $$\begin{array}{c} \left[\begin{array}{c} F_{L}\\ F_{R} \end{array}\right]=\left[\begin{array}{cc} \frac{1}{2} & \frac{1}{\lambda_{s}}\\ \frac{1}{2} & -\frac{1}{\lambda_{s}} \end{array}\right]\cdot \left[\begin{array}{c} m_{cg}\left(\alpha_{cw}+g\sin\theta \right)\\ J_{cg}{\dot{\omega }}_{cw} \end{array}\right], \end{array}$$

where *J*_cg_ is the inertia at the center point of gravity and *α*_cw_ is the rotational acceleration of the center point.

**Figure 16:**
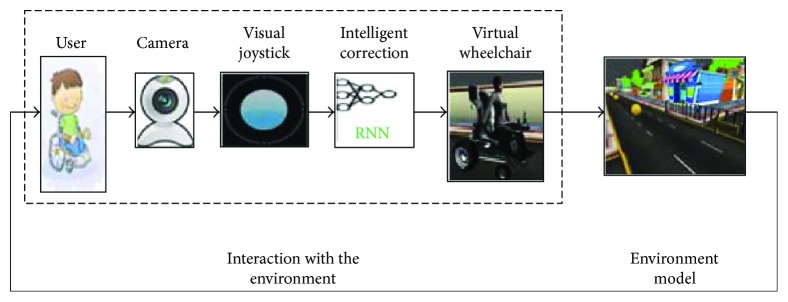
Diagram of virtual simulation.

In order to guarantee the same performance as that of the real wheelchair, these mathematic equations have been implemented on our virtual model using the data in Table 1. The environmental models of the tests have been created using a 3D modeling software (Figure 16).

## 3. System Features

The proposed visual hand controller will be integrated into a real EWC with features presented in Table 1. This specific one has already been used by different researchers [35, 46], in our laboratory, and we still have work to integrate the new control system and functionalities.

It is, basically, composed of 4 wheels, a battery, a joystick, a control chip, and 2 motors. Each motor controls one of the rear wheels.

A joystick is a combination of two potentiometers [47]. Any movement provides analog voltages.  Table 2 shows the voltage map of the joystick movement. A voltage variation in the *x*-axis provides the displacement of the wheelchair to the right or left, and a variation in the *y*-axis provides the speed variation and the movement forward or back.

The relationship between the output of the controller and the voltage given to the motors of the EWC is illustrated by

(18) $$\begin{array}{c} \left[\begin{array}{c} Output1\\ Output2 \end{array}\right]=1.4\times r\left[\begin{array}{c} \cos\theta \\ \sin\theta \end{array}\right]+2.5. \end{array}$$

## 4. Experimental Results

### 4.1. Methodology

In this study, we try to find out the impact of our visual joystick with and without intelligence in a 3D virtual simulator environment. The simulator allows switching various settings in order to test the efficiency of the proposed joystick in various situations.

Usually, it is recommended to simulate the outdoor environments. The dimensions of the environmental elements, particularly the road width, were chosen relatively to those of the wheelchair to be suitable to the actual situations.

### 4.2. Driving Performance Indicators

During the experiment, we took several steps to evaluate the human performance in comparison to the used methods. Some indicators were proposed to evaluate the performance of intelligent wheelchairs [48–50]. These indicators are as follows:

- The joystick movement signals
- Travel time (time taken to accomplish the task)
- The trajectory of the geometric center of the wheelchair
- The number of coin (reference) points to cross (total number is 26)
- Stop number
- Task success (the uncompleted task was considered a failure due to user desertion or failure to reach the final destination)
- The number of collisions during the task
- The average duration of collision that represents the time spent by the system in a contact state with an obstacle
- Mean velocity during motion

These parameters are not independent. That is why an increase in the number of collisions will increase the travel time by adding some maneuvers to overcome the situation.

Other indicators are computed from the proposed intelligent visual joystick such as the average of the amplitude (AR) and angular variation (A*θ*) of the joystick imposed by the user.

(19) $$\begin{array}{c} AR=\frac{\sum_{i=1}^{N}r\left(i\right)}{N},\\ A\theta =\frac{\sum_{i=1}^{N}\theta \left(i\right)}{N}, \end{array}$$

where *N* is the number of samples.

We calculate also the average speed of the EWC during the maneuver using ((20)). This leads to the detection of the state of motion (smoothness/fluidity) of the action on the joystick. Note that a lot of quick changes in direction will lead to high peaks, while a fluid control of the joystick will be translated to low peaks.

(20) $$\begin{array}{c} SR=\frac{\sum_{i=1}^{N}\left(\sqrt{\Delta r{\left(i\right)}^{2}}/\Delta t\left(i\right)\right)}{N},\\ S\theta =\frac{\sum_{i=1}^{N}\left(\sqrt{\Delta \theta {\left(i\right)}^{2}}/\Delta t\left(i\right)\right)}{N}. \end{array}$$

This variable is similar to the jerk indicator used in several studies [51, 52] to describe the comfort or the quality control.

### 4.3. Results

#### 4.3.1. Participants

The aim of this work is to develop a complete method to control a wheelchair that ensures the security by using the least possible resources of sensors and computing power. We focus on the development of an intelligent human-machine interface, which is adaptable to the difficulties encountered by the patient when using the controller of an electric wheelchair.

The therapist must precede systematically either in progressive or in regressive stages, in which some parameters must be considered. Among them, we cite the functional evolution of the gesture and the tremors of the hand.

In this work, we are interested in the functional evolution of the gesture. After approving the clinical protocol (Ref 06.06.2015) by both the University of Tunis and Mohamed Kassab National Institute of Orthopedics, Physical and Functional Rehabilitation Hospital of Tunis, different experimental studies have been launched.

The selection of the participants was made by the team (constituted by the laboratory research team (SIME), educators, and rehabilitation engineers). In our case, we selected three patients with abnormal or involuntary movements and they are physically unable to use their hands to operate the standard joystick.  Table 3 summarizes the different characteristics of the patients and their disabilities.

In our case, the following criteria were considered for patient selection:

- Inclusion criteria
  - Male and female
  - Different patient ages (8 years minimum)
- Exclusion criteria
  - Pregnant women
  - Persons deprived of liberty
  - Inclusion in another research protocol submitted for consent
  - Voluntary withdrawal by the patient
  - Stopping of the study based on the explicit decision of the doctor

The level V means that the children have physical deficiencies that limit voluntary control of movement and the ability to maintain the head and neck position against gravity. The patient cannot sit or stand independently, even with adaptive equipment, and cannot independently walk, though he/she may be able to use powered mobility.

Functional independence measure (FIM) allows assessing the patient's dependence in terms of motor, cognitive, psychological, and behavioral capacity by measuring the limits and the need for assistance. It possesses an ordinal scale of 18 items, including communication and social cognition.

#### 4.3.2. Calibration of the Joystick

**Figure 17:**
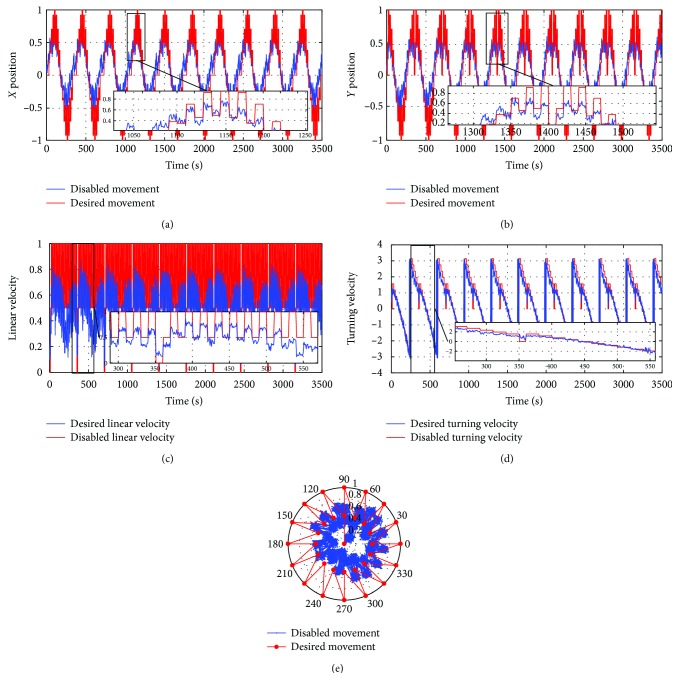
The data recorded during the data collection phase using the visual joystick without an intelligent corrector: (a) the displacement along the *x*-axis; (b) the displacement along the *y*-axis; (c) linear velocity; (d) turning velocity; (e) the movement in the polar coordinate.

Figure 17 shows the data given by the first patient during the data collection phase. It, respectively, represents the displacements along the *y*-axis, the displacements along the *x*-axis (blue color), and the desired signal (red color).

The superposition of the signals shows the hand movement limitations of this patient in any direction when compared to the referenced signal.

**Figure 18:**
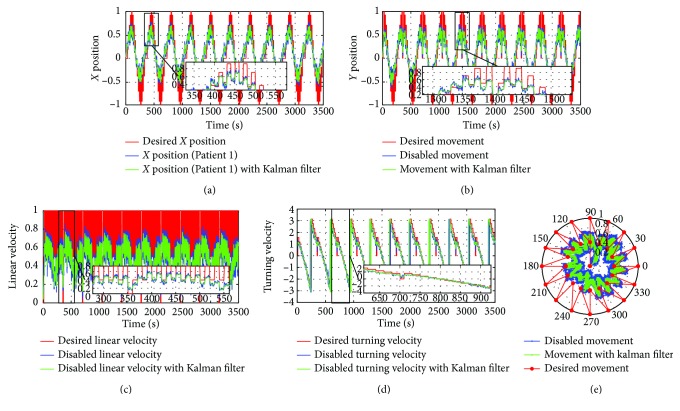
The data recorded with the Kalman filter effect: (a) the displacement along the *x*-axis; (b) the displacement along the *y*-axis; (c) linear velocity; (d) turning velocity; (e) the movement in the polar base.

In this figure, we can see the amount of noise, which affects the patient's test, and therefore, we add the Kalman filter whose effect appears in Figure 18.

After filtering the patient's control signals, we will correct the gaps appearing between the signals and the reference one through the RNN.

**Figure 19:**
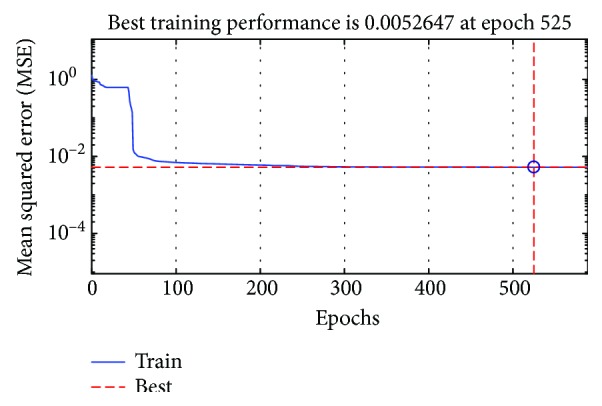
The mean square error of the training set.

To do this, we have trained the recurrent neural network with the data recorded by the first patient with the Kalman filter effect and the desired data until minimizing the mean square error.  Figure 19 shows that the RNN fits well with the difficulties encountered by the patient during the operation of the visual joystick.

**Figure 20:**
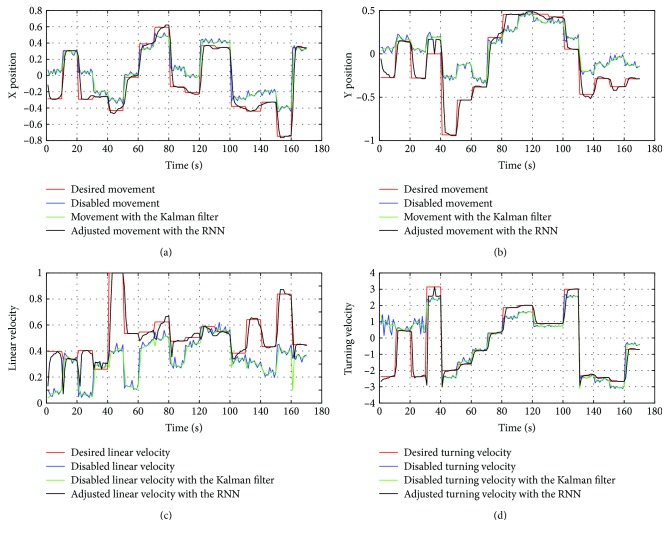
The data recorded in the test set using the visual joystick with an intelligent corrector: (a) the displacement along the *x*-axis; (b) the displacement along the *y*-axis; (c) linear velocity; (d) turning velocity.

The evaluation results of the recurrent neural network are performed with a new set of data (referred as the test set), in which the patient is asked to track the movement of the pink cross that appears on the screen in other positions than the tracking phase.  Figure 20 illustrates these results.

#### 4.3.3. Trajectory Analysis

**Figure 21:**
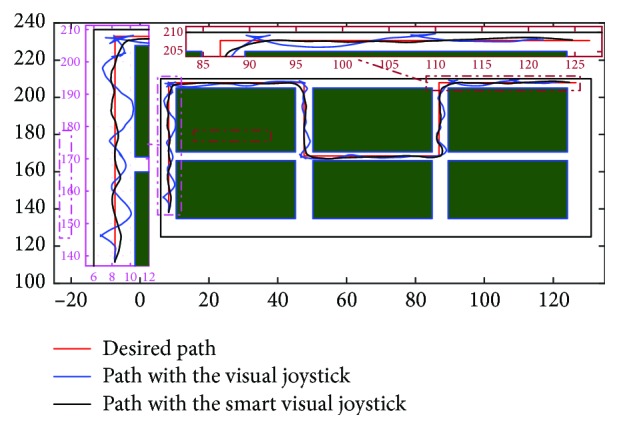
Comparison between the trajectories of the first patient with and without the proposed intelligent visual joystick.

**Figure 22:**
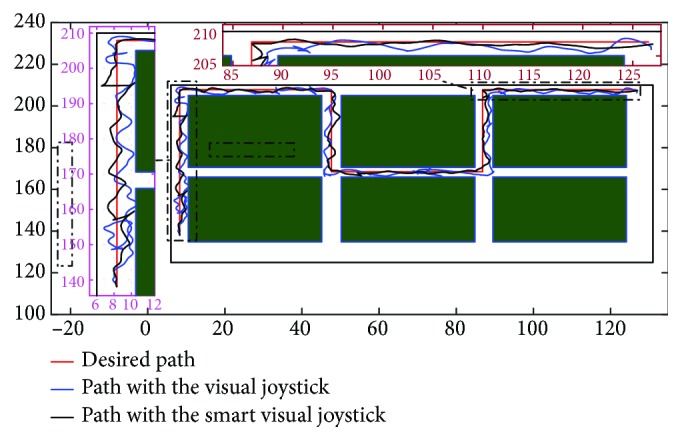
Comparison between the trajectories of the second patient with and without the proposed intelligent visual joystick.

**Figure 23:**
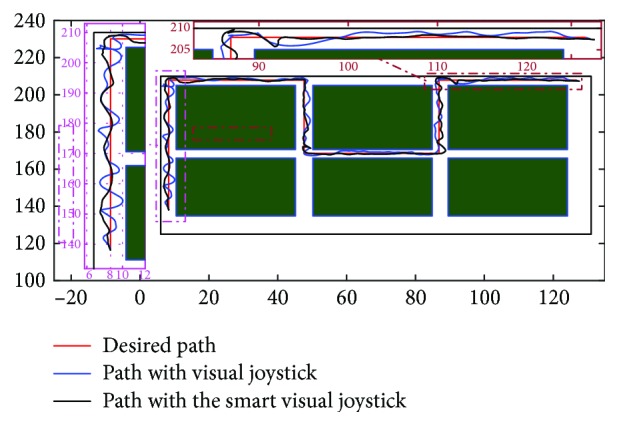
Comparison between the trajectories of the third patient with and without the proposed intelligent visual joystick.

The test protocol was included in the user rehabilitation session. In two steps, with and without intelligence, some training paths are executed in order to start adapting the users with the used visual joystick. In fact, a repetition of five paths at a speed range (0–1.7 m/s) was made in the test environments. The choice of making two kinds of driving tests (with and without intelligence) was motivated to allow the user to go native with the intelligence effect over a long time instead of a split time use. To do this, we ask the patient to follow a targeted trajectory that appears on the 3D simulator. These paths must be reached in order to be validated. The trajectories of the three users (for the last trial) are illustrated in Figures 21, 22, and 23.

The computation of the Hausdorff and Frechet distance can give us an idea of the gap between the trajectory made by the patient and the one loaded in the reference (Table 4). These algorithms estimate the geometric similarity of trajectories using metric paths [53]. They return a nonnegative number (a distance). Two paths are identical if their distance is zero, and large distances indicate dissimilarity. To properly analyze a path, one must select a suitable reference structure to which all paths will be universally aligned using the rotations determined by the best fit of root mean square deviation (RMSD).

The driving performance indicators mentioned above are illustrated in Table 5 where we conclude that the three patients have found difficulty in navigation, and that difficulty is quite clear in the number of collisions measured and incomplete tasks. We also notice that patients are increasingly tired and it is shown by the loss of time regardless of the browsing time or the static time (no movement).

The table also shows the improvement after the activation of the smart visual joystick. The improvement is in terms of numbers of target points by which patients have to pass through, the total test time and movement, the distance traveled by patients, the numbers of collisions, and the incomplete tasks.

#### 4.3.4. Amplitude and Average Physical Speed of the Proposed Joystick

**Figure 24:**
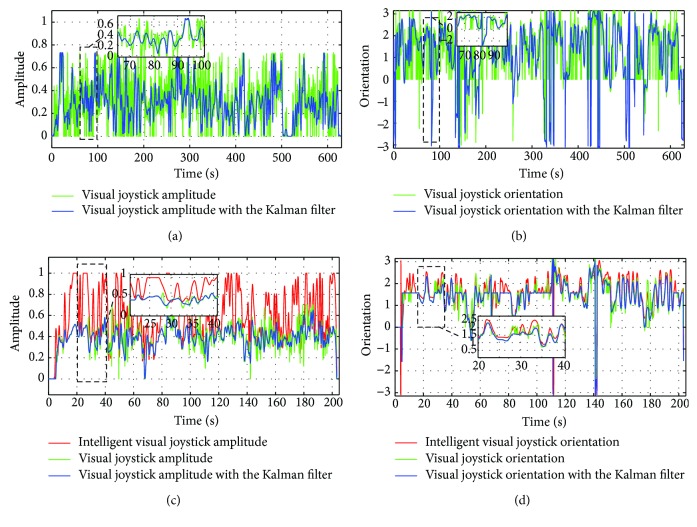
The data recorded by a patient during the maneuver: (a) the visual joystick amplitude without a recurrent neural network corrector; (b) the visual joystick orientation without a recurrent neural network corrector; (c) the visual joystick amplitude with a recurrent neural network corrector; (d) the visual joystick orientation with a recurrent neural network corrector.

Table 6 presents the average amplitude of the visual joystick control with and without an intelligent corrector measured during the maneuver, for example, Figure 24. It is noted that, for most of the patients, adding an intelligent corrector increases these amplitudes. This allows us to conclude that the corrector declines significantly the time taken to accomplish the task.

Average physical speed of the joystick SR and S*θ*, shown in Table 6, is linked to signal variations (amplitude and orientation) of the visual joystick with and without intelligence during the navigation.

**Figure 25:**
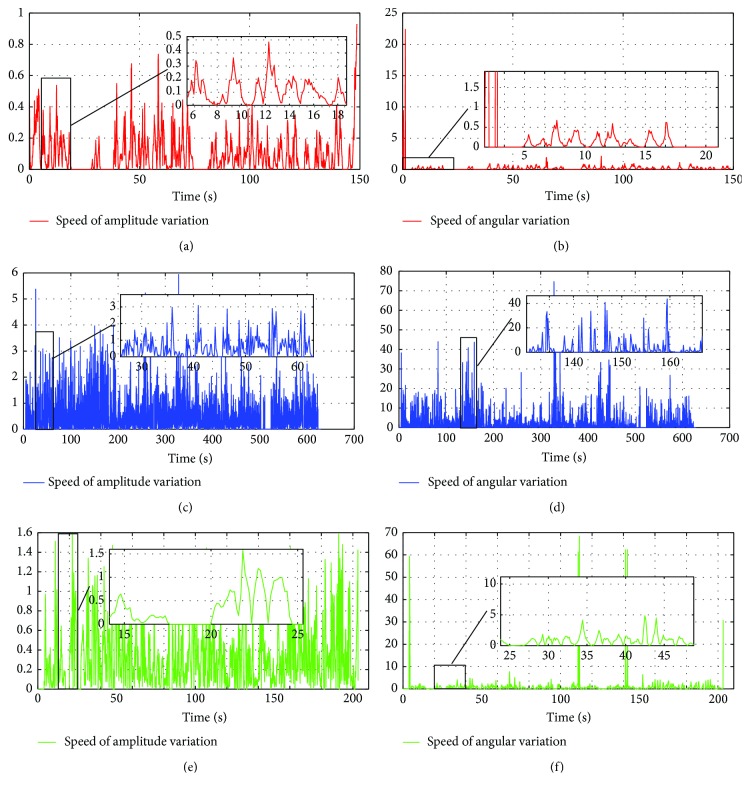
The average physical speed of the visual joystick: (a) the speed amplitude variation made by an expert user without an intelligent corrector; (b) the speed orientation variation made by an expert user without an intelligent corrector; (c) the speed amplitude variation made by the first patient without an intelligent corrector; (d) the speed orientation variation made by the first patient without an intelligent corrector; (e) the speed amplitude variation made by the first patient with an intelligent corrector; (f) the speed orientation variation made by the first patient with an intelligent corrector.

In Figure 25, we present the average physical speed of the visual joystick with and without the calibration mode according to the path of the first patient. Generally, the recurrent neural network corrector allows decreasing the incorrect handling of the visual joystick. This leads to a reduction in the variations of the control signal that permits more flexible and smoother driving of the wheelchair.

The user moves the joystick significantly less to achieve the same tasks, therefore using less effort. However, it can be seen that in the majority of cases, the collaborative system requires significantly less movement than the traditional method, to perform the same critical tasks.

## 5. Discussion

Various appropriate considerations must be cited in order to facilitate the use of our proposed method in the wheelchair driving task for people with motor disabilities. The temporal and the average physical speed of the joystick applied to the resulting data from the patient and the number of collisions shows that the new strategy acts in general positively on the control. Hence, the runtime is decreased significantly. In addition, the patient operates with accurate magnitudes. In addition, the orientation and the control signal variations are less important on the wheelchair. Other indicators, such as the Hausdorff distance and Frechet distance of the trajectories, show big differences in control behavior when using the suggested method.

Note that doctors confirm the efficiency of the developed intelligent visual joystick for the users during tests either to reduce the runtime or to enhance controls.

In this work, we executed a 3D simulator with an intelligent control to drive the electric wheelchair with real parameters. The design of a real instrumented PW to have an intelligent visual joystick became so possible and easy to be implemented, due to the high level of technology. This implementation has been already done and was successfully tested on a panel of healthy people in the laboratory.

However, when testing the real prototype on patients with disabilities, some practical safety problems appeared. In this case, the simulator allows a great flexibility in operation such as easy data gathering, as well as completely safe tests.

After finishing the test using the proposed system, the participants were interviewed to investigate their level of satisfaction. To obtain more details about their opinions, the System Usability Scale of the participants has been used after driving sessions. There are many studies [54–57] using the System Usability Scale (SUS) [58] which is a ten-item scale for administering after usability tests.

Three participants were satisfied with the proposed intelligent visual joystick. The participants replied with average satisfaction rates of 96%, 92%, and 84% for each user, respectively. During the interviews, all participants approved that they experienced less physical damages and required less effort to complete the navigation when using the intelligent visual joystick.

## 6. Conclusion and Perspectives

Many interface devices have been well described in the literature for controlling an electric wheelchair. These interfaces have shown encouraging results. However, they are not advanced enough to conclude on their actual contribution. In addition, they allow controlling the wheelchair only for four different commands (forward, backward, left, and right) at a predefined speed.

Thereby, we created a new control interface of an electric wheelchair based on hand gestures. This control differs from the other control technologies allowing movement in different directions at variable speed and subsequently a similar control of classical joystick that provides driving flexibility for the user.

On the other hand, it adapts to different types of disabilities through the recurrent neural network introduced into the system, which makes it a smart joystick.

In order to compare the driving performance with and without our smart control system, we referred to some parameters such as processing time, distance traveled, and the number of collisions. Simulation results, performed on a disabled person of Mohamed Kassab National Institute of Orthopedics, Physical and Functional Rehabilitation Hospital of Tunis, validate this new visual control interface.

This work can be validated further by passing from the 3D simulator control to the real driving wheelchair and by testing this control on a large number of disabled people with different pathologies.

## Figures and Tables

**Table 1:** The features of the proposed intelligent wheelchair.

Parameters of the wheelchair		A processing device (Raspberry pi II Model B)		Camera (LifeCam Cinema H5D-00013)
Height	89 cm	Price	$39.99	720p HD video up to 30 fps
Width	61 cm	Chip	Broadcom BCM2836	Autofocus
Frame weight with batteries	58 kg	Processor	ARMv7 quad-core	High-precision glass element lens
Load capacity	110 kg	Processor speed	900 MHz	TrueColor technology
Linear velocity	8 km/h	Voltage and power draw	650 mA at 5 V	360-degree rotation
Ø front wheels	20 cm	GPU	Dual-Core Video Core IV Multimedia Co-Processor	Wide-angle lens
Ø rear wheels	30 cm	Size	85 × 56 mm	Widescreen 16 : 9 format
Stopping distance	1 m	Memory	1 GB SDRAM at 400 MHz	Connectivity (USB 2.0)
Noise	65 dB	GPIO	40	
Battery life	20 km	USB 2.0	4	
Battery	2 × 12 V 28 Ah	Ethernet	10/100 Mb Ethernet RJ45 Jack	
Engine power	2 × 220 W 24 V	Audio	Multichannel HD audio over HDMI, analog stereo from 3.5 mm headphone jack	

**Table 2:** Operation volts of the intelligent wheelchair.

	Output1	Output2
Stop	2.5 V	2.5 V
Forward movement	2.5 V	2.5 V~3.9 V
Back movement	2.5 V	1.1 V~2.5 V
Right turn	2.5 V~3.9 V	2.5 V
Left turn	1.1 V~2.5 V	2.5 V

**Table 3:** Characteristics of patients.

Characteristics	Patient I	Patient II	Patient III
Sex, age, mass (kg)	Female, 24, 60	Male, 15, 54	Male, 16, 45
Diagnosis	Cerebral palsy	Posttraumatic tetraplegia	Cerebral palsy
Motor disability and clinical symptoms	Spastic tetraplegia	Spastic tetraplegia C5	Dyskinetic tetraplegia
Handedness	Right-handed	Left-handed	Right-handed
Functional level	GMFCS^∗^: V FIM: 86	FIM: 82	GMFCS: V FIM: 89
Powered wheelchair driver	None	None	None

^∗^The functional level of the GMFCS (Gross Motor Function Classification System) contains five different levels (I–V) for children and young people with cerebral palsy. It is helpful because it provides families and clinicians the clear description of a child's current motor function. In addition, it gives an idea about what equipment or mobility assistance a child may need in the future, for example, crutches, walking frames, or wheelchairs.

**Table 4:** Comparison between the trajectories of the users.

		Hausdorff distance	Frechet distance
The similarity between the desired path and path made by an expert		12.8013	12.9334
Patient I	Similarity between the desired path and visual joystick path	13.2911	13.3452
	Similarity between the desired path and intelligent visual joystick path	13.0561	13.0707

Patient II	Similarity between the desired path and visual joystick path	13.3055	13.3701
	Similarity between the desired path and intelligent visual joystick path	13.0033	13.1265

Patient III	Similarity between the desired path and visual joystick path	13.3207	13.3444
	Similarity between the desired path and intelligent visual joystick path	13.1595	13.2172

**Table 5:** Performance indices from the users' paths.

		Path length (m)	Time (sec)	Speed (m/sec)	Number of collisions	Uncompleted task	The average duration of collision (s)	The number of coin points to cross	Stop number
Path made by an expert		248.509	148.638	1.6719	0	0	0	26	0

Patient I	Disabled path	322.871	628.463	0.5710	7	3	3.228	14	9
	Intelligent visual joystick path	261.070	203.297	1.3647	0	1	0	21	3

Patient II	Disabled path	297.6955	670.648	0.4700	5	2	3.461	17	4
	Intelligent visual joystick path	289.7011	262.4178	1.1882	3	0	2.874	20	2

Patient III	Disabled path	410.6635	949.965	0.5305	26	4	4.843	12	14
	Intelligent visual joystick path	306.1023	319.034	1.0563	5	1	3.451	17	3

**Table 6:** Performance indices from the joystick signals.

		AR	A*θ*	SR	S*θ*
Path made by an expert		0.7635	1.5721	0.0870	0.1981

Patient I	Visual joystick path	0.3235	1.1534	0.5039	2.2283
	Intelligent visual joystick path	0.5946	1.5450	0.3425	0.9785

Patient II	Visual joystick path	0.2934	1.7811	0.2849	1.6488
	Intelligent visual joystick path	0.5902	1.6058	0.1149	0.8327

Patient III	Visual joystick path	0.4882	1.0283	0.5688	3.0052
	Intelligent visual joystick path	0.6034	1.4028	0.2084	0.9911
